# Effects of polymorphism of *myostatin* and *fatty acid-binding protein 4* genes on the chemical composition of meat in cull female *Aceh* cattle

**DOI:** 10.14202/vetworld.2020.1334-1343

**Published:** 2020-07-14

**Authors:** Al Azhar, Muslim Akmal, Muhammad Hambal, Mustafa Sabri, Teuku Shaddiq Rosa

**Affiliations:** 1 Department of Biochemistry , Faculty of Veterinary Medicine, Universitas Syiah Kuala, Banda Aceh, Aceh 23111, Indonesia; 2 Department of Histology , Faculty of Veterinary Medicine, Universitas Syiah Kuala, Banda Aceh, Aceh 23111, Indonesia; 3 Department of Parasitology , Faculty of Veterinary Medicine, Universitas Syiah Kuala, Banda Aceh, Aceh 23111, Indonesia; 4 Department of Anatomy , Faculty of Veterinary Medicine, Universitas Syiah Kuala, Banda Aceh, Aceh 23111, Indonesia; 5 Master Program of Veterinary Public Health, Faculty of Veterinary Medicine, Universitas Syiah Kuala, Banda Aceh, Aceh 23111, Indonesia

**Keywords:** *Aceh* cattle meat, ash, cholesterol, fat, polymerase chain reaction-restriction fragment length polymorphism, protein

## Abstract

**Aim:**

This study aimed to investigate the association of single nucleotide polymorphism of the *myostatin* (*MSTN*) and *fatty acid-binding protein 4* (*FABP4*) genes on the total water, ash, fat, protein, and cholesterol contents of sirloin (*gluteus medius* muscle) and silverside (*biceps femoris* muscle) meats of cull female *Aceh* cattle.

**Materials and Methods:**

This analysis covered a total of 27 cull female *Aceh* cattle slaughtered at the Animal Slaughterhouse of Banda Aceh that was purposively selected based on hair color referred to the criteria described in the Decree of Ministry of Agriculture of the Republic of Indonesia. Genomic DNA was extracted from 25 mg of fresh meat using the spin column method before subjected to a polymerase chain reaction amplification using primer sets specific for 1346-bp and 275-bp fragments of *MSTN* and *FABP4*, respectively. A 4-h digestion reaction was done separately for the *MSTN/HaeIII* and *FABP4/NlaIII* loci genotyping. The total protein, ash, and fat of the meat were measured using the Indonesian National Standard (SNI) methods whereas its cholesterol content was determined using the AOAC method. The association between each polymorphism and the variation in meat chemical parameters was analyzed using the Pearson correlation test.

**Results:**

The results showed that the *MSTN/HaeIII* locus was polymorphic in *Aceh* cattle, but the *FABP4/NlaIII* locus was monomorphic. Meat chemical parameters were not influenced by different commercial cuts and *MSTN* *genotypes*, showing that there was no association between different commercial cuts, cattle hair colors, and *MSTN/HaeIII* and *FABP4/NlaIII* markers with the meat chemical parameters in *Aceh* cattle.

**Conclusion:**

These results suggest that focusing on the novel effects of *MSTN* and *FABP4* gene polymorphisms on meat production traits might not be useful for marker-assisted selection in *Aceh* cattle.

## Introduction

Interaction of one or more genetic polymorphisms with environmental factors is responsible for the inheritance of many economically desired traits of domesticated animals [1] such as higher production (dressing percentage, meat quality, and milk result) [2], reproduction [3], and disease resistance [4], as well as better adaptation to moisture, a hot tropical climate [5], low-quality food, and traditional farming [6]. These traits of economic importance, however, are not concurrently inherited in animals. Indigenous cattle, for example, are well known for good adaptation and low maintenance price, but have slow growth, delayed puberty, and low production compared to exotic cattle [7]. Slow growth, delayed puberty, and low production are traits generally attributed to *Bos indicus* (zebu) cattle [8]. The occurrence of the zebu genotype is also responsible for low meat tenderness [9] and texture [10], but higher fat content [11], three of the most important parameters of meat quality in the beef industry. Searching for the genetic basis of the desired phenotypic variations followed by integrating the data obtained in the currently used conventional breeding selection might shorten the selection process of Indonesian beef cattle.

Several genetic factors have been identified to be associated with varying meat nutritional quality trait. Among them, myostatin (*MSTN*) and *fatty acid-binding protein 4* (*FABP4*) genes are the most interesting candidate factors. Inconsistent relationships may be found between these potential genetic markers and the meat quality of several cattle populations [12]. While some of the previously identified 20 polymorphisms (due to nucleotide deletion, insertion, or substitution) of *MSTN* are known to be linked to increased muscle [13,14], polymorphisms found in either the exon or intron of *FABP4* have been shown to affect bovine backfat thickness [15], marbling, and carcass weight [16]. In Indonesia, unfortunately, there are limited information about meats chemical composition and nutritional value of local beef cattle. Furthermore, are not available scientific data about the potential genetic variations related to meat chemical and physical parameters. Although the diversity of exon-3 of *MSTN* has been studied in Indonesian *Madura* cattle[17], a previous study by Nugroho *et al*. [18] in *Bali* cattle is the only one that shows positive effects of *MSTN* polymorphism on the chest girth, weaning weight, and chest circumference.

At present, no research has addressed the effect of single polymorphism of *MSTN* and *FABP4* on meat chemical parameters in *Aceh* cattle, a genetic resource of Indonesian beef cattle native to Aceh Province which originated from the cross-breeding of Indonesian local cattle (*Bos sondaicus*), Java cattle (*Bos javanicus*), and zebu cattle (*Bos indicus*) [19,20]. Naturally *Aceh* cattle have different hair colors, but only *Aceh* cattle have brick red hair color which fulfills the criteria mentioned in the Indonesian National Standard. We hypothesized the *MSTN* and *FABP4* polymorphisms, different commercial cuts, and cattle hair colors might have some effects on meat chemical parameters and the physical quality of *Aceh* cattle, but there is no scientific information to prove this assumption.

Therefore, this study aimed to investigate the effect of *MSTN* and *FABP4* polymorphisms on the chemical parameters of sirloin (*gluteus medius* [GM] muscle) and silverside (*biceps femoris* [BF] muscle) meats of *Aceh* cattle with different hair colors. Information obtained might provide a scientific basis for setting *Aceh* cattle with brick red hair color as the Indonesian National Standard criteria and might have some benefits to the existing cattle breeding selection programs in Indonesia.

## Materials and Methods

### Ethical approval

All protocols used in this study have been approved by the Faculty of Veterinary Medicine Committee of Animal Ethics, Universitas Syiah Kuala, Banda Aceh (Ref: 28/KEPH/II/2018).

### Samples collection

This study was conducted from May 2018 to March 2019. Fresh sirloin (GM muscle) and silverside (BF muscle) meats, 250 g each, of 27 cull female *Aceh* cattle aged 8-12 years old with different hair colors (three light brown, three red brown/brick red, five grayish black, four black, six straight yellow, three grayish yellow, and three white) were purposively purchased from the Animal Slaughterhouse of Banda Aceh. This was to accommodate the naturally existing hair color variations among *Aceh* cattle herds as mentioned in the Decree of the Ministry of Agriculture of the Republic Indonesia Number 54/Permentan/OT.140/10/2006. The meats were kept cool (4°C), brought to the Faculty of Veterinary Medicine Laboratory of Research at Universitas Syiah Kuala, and stored at −20°C before examination. Molecular characterizations were performed on 27 sirloin meat samples collected and chemical analysis was performed on 40 (20 sirloin and 20 silverside) out of 54 meat samples due to limited research funding.

### Meat chemical analysis

Using the commercial service at the Center for Agro-based Industry of Bogor, the total water, ash, fat, and protein contents of the meat samples were determined using the Indonesian National Standard (SNI) test methods of food and beverage [21], whereas meat cholesterol content was measured using the AOAC method [22]. Meat moisture was analyzed using the SNI protocol No. 01-2891-1992 point 5.1 (oven method). Here, 1-2 mg of meat sample was put in a pre-weighed closed bottle, and dried in an oven (105°C) for 3 h. The sample was cooled and reweighed. The procedure was repeated until a constant weight was achieved. Water content was quantified as a percentage of the sample mass [21].

The SNI method No. 01-2891-1992 point 6.1 (dry method) was used to determine meat ash content. Briefly, a porcelain cup was pre-conditioned in a furnace at an ashing temperature of 550°C for 30 min, cooled at room temperature, and weighed. Meat samples, 1-2 g, were individually weighed using this porcelain cup, placed in a furnace, and dried to charcoal before an ashing step at 550°C. The porcelain cup containing ash was cooled to room temperature in a desiccator and weighed. Total ash content was quantified as a percentage of the sample mass [21].

Meat protein content was measured using the SNI method No 01-2891-1992 point 7.1 (Kjeldahl method). Here, 0.51 g of meat sample was combined with 2 g of selenium and 25 mL of concentrated H_2_SO_4_, and heated for 2 h. The mixture was cooled and diluted to 100 mL with distilled water. Five milliliters of aliquot mixture were mixed with 5 mL of 30% NaOH and 3-5 mL of phenolphthalein indicator and distillated for 10 min with 2% boric acid. The volume of 0.01 N HCl solution used to neutralize distillate was measured to determine the nitrogen percentage. The protein content was calculated by multiplying the nitrogen percentage with 6.25 [21].

The Soxhlet method (SNI No. 01-2891-1992 point 8.2) was used to measure meat fat content. In brief, approximately 1-2 g of meat samples were put into a cotton-lined paper sleeve and dried for 1 h using an oven heated to <80°C. The dry meat was put into a Soxhlet apparatus connected with a fat jar containing a pre-weighed boiling stone. The fat was extracted using a hexane for 6 h. After distilling the hexane, the extracted fat was dried in an oven (105°C), cooled and weighed. Fat content was quantified as a percentage of the sample mass [20].

Meat cholesterol content was determined using gas chromatography according to the Association of Official Analytical Chemists (AOAC, USA) No. 994.10 [21]. Briefly, 2 g of fresh meat (W1) was saponified with 40 mL of 95% ethanol and 8 mL of 50% potassium hydroxide for approximately 70 min at 60°C. The mixture was combined with 60 mL of 95% ethanol, incubated for 15 min, and cooled in a closed flask at room temperature. The non-saponified fraction was extracted 3 times using 100 mL of toluene (*V_1_*), mixed with 110 mL of 1M KOH by vigorous shaking for 10 min, and incubated at room temperature to allow layers formation. The toluene layer was mixed with 40 mL of 0.5 M KOH and washed ≥3 times with distilled water. The clear toluene layer formed was poured into a new 125 mL Erlenmeyer flask, added with 2 g of Na_2_SO_4_, and incubated at room temperature for ≥15 min. The extract, 25 mL (*V_2_*), was evaporated to dryness on a rotary evaporator at 40±3°C, and mixed with 3 mL of acetone. After evaporating the extract to dryness again, the residue was dissolved in 3 mL of dimethylformamide (*V_3_*). Standard aliquots (0.0025-0.2 mg/mL), 1 mL each, were mixed with 2.0 mL of hexamethyldisilane, incubated at room temperature for 15 min, and then mixed with 1 mL of 5α-cholesterol internal standard solution (1 mg/mL in heptane). After centrifugation for 2 min, the heptane layer was collected. Standards and test solutions, 1 μL each, were injected to a gas chromatograph. Peak areas of 5α-cholestane and cholesterol were determined using height-weight measurements. Standard response ratio was calculated by dividing cholesterol peak area by internal standard peak area. The ratio response of the four highest standards was plotted against cholesterol concentrations. The amount of meat sample portion per milliliter (in grams) was determined by multiplying the ratio of meat sample examined and the volume of toluene used with the ratio of aliquot taken to dryness and the volume of dimethylformamide used to solve residue (*W_1_*/*V_1_* x *W_2_*/*V_2_*). Meat cholesterol content (mg) was determined by dividing the amount of cholesterol in the meat sample examined based on a standard curve by the amount of sample portion per milliliter [22].

### DNA extraction

Genomic DNA was isolated from each sirloin meat sample at room temperature using a PureLink^™^ Genomic DNA Mini Kit (Invitrogen Life Technologies, USA) following protocol provided by the manufacturer. In brief, 20-25 mg of fresh meats were minced, immersed in digestion buffer mix, and incubated at 55°C for 2 h with occasional vortexing. After spinning for 3 min at 16,000× g, supernatant was mixed with 20 μL of RNase A and incubated for 2 min. The lysate was mixed with 200 μL of genomic lysis/binding buffer, added with 200 μL of absolute ethanol, and mixed by short vortexing. The entire mixture was transferred to a spin column in a collection tube and spun at 1000× g for 1 min. After replacing the collection tube, 500 μL of wash buffer-1 was added, and the column was respun at 10,000× g for 1 min. This washing step was repeated using wash buffer-2 and 3-min spinning at 16,000× g. Genomic DNA was eluted from the spin column by 1 min incubation with 50 μL of elution buffers followed by spinning at 16,000× g for 1 min. The quality of DNA extract was checked by electrophoresis on a 1% agarose/1×TAE gel stained with a SYBR™ Safe (Invitrogen Life Technologies, USA) stain using a 100-bp ladder as a molecular size marker, and visualized using a digital imaging system (Bio-Rad, USA). Purified DNA extract was stored at −20°C [23].

### Genotyping

Polymorphism of *MSTN* and *FABP4* was examined with the polymerase chain reaction-restriction fragment length polymorphism (PCR-RFLP) approach. Primer sequences, PCR conditions and restriction enzymes used are presented in Table-1 [16,18,24]. The PCR reaction mixture (25 μL) was prepared by adding 3 μL of template DNA (5-10 pg), 12.5 μL of PCR-master mix (Invitrogen Life Technologies, USA), forward and reverse primers (1 μL each), and 7.5 μL of nuclease-free water. The DNA amplification was done in a Bio-Rad (USA) thermal cycler. After amplification, 5 μL of PCR products were added with 15 units of the corresponding restriction enzyme, and incubated at 37°C for 4 h. The digestion products were analyzed by electrophoresis on 1.5% agarose/1×TAE gel (Invitrogen Life Technologies, USA) stained with a SYBR™ Safe stain using a 100-bp ladder as molecular size marker. The electrophoresis was run at 80V for 1.5 h and visualized using a digital imaging system (Bio-Rad, USA) [23].

**Table-1:** Fragment size, PCR primers and conditions, and restriction enzymes used in the analysis of *MSTN* and *FABP4* polymorphisms.

Gene	Fragment size (bp)	PCR primers (5′ to 3′)	PCR conditions	Restriction enzyme	Reference
*MSTN*	1,346	F: 5′CCCTACAGAGGCCACTTCAA3′ R: 5′CTCGCTGTTCTCATTCAGATC3′	94°C 3′, (94°C 3 s, 63°C 30 s, 72°C 1′) 39 cycles, 72°C 10′	HaeIII	[24] [18]
*FABP4*	565	F: 5′ACCCCTATGATGCTATTCCACA3′ R: 5′ATACGGTTCACATTGAGAGGGA3′	95°C 4′, (94°C 1′, 60°C 1′,72°C 1.5 min^−1^) 35 cycles, 72°C 5′	NlaIII	[16]

### Statistical analysis

Based on the DNA pattern, genetic polymorphism was tested using the formula: PiCi = 1 - Σp2ij. PiCi is the polymorphic information contained for the i^th^ locus and pij is frequency of the j^th^ allele for the i^th^ locus. A Chi-square test was used to determine the agreement of allele distribution to the Hardy-Weinberg equilibrium. Data allele and genotype frequencies were analyzed by SPSS software version 23 for Windows (IBM, USA). While difference in meat composition between different commercial cuts was determined using a one-way ANOVA, the relationship between meat chemical parameters and gene polymorphism was analyzed using Pearson’s correlation test.

## Results

### Meat chemical composition

The meat chemical parameters in the two commercial cuts of the cattle analyzed were comprised of water, ash, protein, fat, and cholesterol, as presented in Table-2. Silverside meats of *Aceh* cattle tended to have higher moisture, ash, protein, and cholesterol contents than sirloin meats. Sirloin meats, on the other hand, slightly contained more fat than silverside meats. The differences in meat chemical parameters observed, however, were not significant (p>0.05).

**Table-2:** Meat chemical parameters (±SD) of two commercial cuts of *Aceh* cattle.

Chemical parameters	Commercial cut		Average

	Sirloin	Silverside	
Water (%)	69.65±4.60^ns^	71.50±4.64^ns^	70.57±4.66
Ash (%)	0.96±0.09^ns^	0.97±0.10^ns^	0.96±0.09
Protein (N x 6.25%)	16.93±2.56^ns^	17.48±2.72^ns^	17.20±2.62
Fat (%)	3.62±2.87^ns^	2.52±1.71^ns^	3.07±2.40
Cholesterol (%)	71.62±17.03^ns^	72.18±28.14^ns^	71.90±22.96

ns=differences presented in the same row were not significant

Meat chemical parameters of *Aceh* cattle with different hair colors are listed in Table-3. The water, ash, protein, fat, and cholesterol contents in the meats of *Aceh* cattle with black hair were not significantly different from *Aceh* cattle with grayish black hair, straight yellow hair, white hair, and brick red hair.

**Table-3:** Meat chemical parameters (±SD) of *Aceh* cattle with different hair colors.

Chemical parameters	Cattle hair color				

	Black	Grayish black	Straight yellow	White	Brick red
Water (%)					
Sirloin	69.40±6.45^ns^	67.63±5.57^ns^	70.57±5.28^ns^	69.73±0.51^ns^	70.43±4.55^ns^
Silverside	70.90±6.20^ns^	68.93±5.45^ns^	72.00±4.36^ns^	72.97±4.69^ns^	72.65±4.61^ns^
Ash (%)					
Sirloin	0.96±0.16^ns^	0.96±0.04^ns^	0.96±0.07^ns^	0.95±0.16^ns^	0.97±0.11^ns^
Silverside	0.87±0.10^ns^	0.95±0.06^ns^	0.99±0.09^ns^	0.99±0.11^ns^	1.00±0.11^ns^
Protein (%)					
Sirloin	16.87±4.60^ns^	17.28±1.15^ns^	17.05±2.14^ns^	16.03±3.54^ns^	17.13±2.98^ns^
Silverside	16.33±3.96^ns^	18.10±2.05^ns^	17.72±1.77^ns^	16.33±4.68^ns^	18.20±2.92^ns^
Fat (%)					
Sirloin	2.42±0.78^ns^	4.89±2.76^ns^	4.89±4.32^ns^	2.13±0.33^ns^	2.44±1.44^ns^
Silverside	3.24±0.56^ns^	4.36±2.81^ns^	1.50±0.89^ns^	1.50±0.37^ns^	2.42±0.97^ns^
Cholesterol					
Sirloin	64.10±8.82^ns^	79.38±21.57^ns^	75.75±19.89^ns^	65.37±22.65^ns^	67.98±9.53^ns^
Silverside	60.20±8.88^ns^	69.53±20.51^ns^	74.95±18.95^ns^	97.53±64.26^ns^	60.63±14.24^ns^

ns=differences presented in the same row were not significant

### Polymorphism of *MSTN* and *FABP4* and meat chemical composition

**Figure-1:**
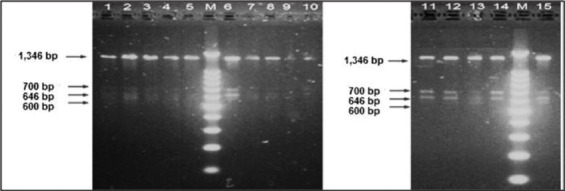
Results of agarose gel electrophoresis of myostatin reaction restriction fragment length polymorphism fragments (1346, 700, 646, and 600 bp). Lane M, 100 bp ladder. Lane 01-05, *Aceh* cattle examined [23].

**Figure-2:**
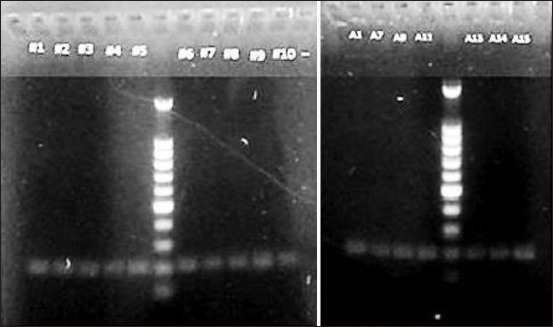
Results of agarose gel electrophoresis of *FABP4* restriction fragment length polymorphism fragment (200 bp). Lane M, 100 bp ladder. Lane 01-15, *Aceh* cattle examined.

The results from PCR-RFLP experiment using the HaeIII restriction enzyme on 27 sirloin meat samples revealed that the *MSTN* is polymorphic in *Aceh* cattle as shown by the occurrence of AA, AB, and BB genotypes in the population [23]. The *FABP4*, on the other hand, is monomorphic in *Aceh* cattle by the presence of a single uncut AA genotype. The results of PCR-RFLP of *MSTN* and *FABP4* fragments from several samples are presented in Figure-1 and 2 [23], respectively.

As shown in Figure-1, the PCR-RFLP analysis using the HaeIII enzyme showed that the 1346 bp *MSTN* fragment was cut into 700 and 646 bp fragments (AA), 700, 646, 618, and 28 bp fragments (AB) or 618 and 28 bp fragments (BB). With a polymorphism degree of 0.51 (Table-4) [23], the *MSTN genotype* distribution in the *Aceh* cattle population agreed well with the Hardy-Weinberg equilibrium (*χ*^2^=0.55). The respective frequency of allele A and B was 0.45 and 0.55.

**Table-4:** *MSTN* allele and genotype frequencies and polymorphism in *Aceh* cattle*.

Total genotype		Frequency		Polymorphism degree	Hardy-Weinberg Equilibrium (χ^2^ test)
	
Observed	Expected	Genotype	Allele		
AA=5	AA=4.1	AA=0.18	A=0.45	0.51	χ^2^=0.55
AB=11	AB=12.1	AB=0.41	B=0.55		
BB=11	BB=10.8	BB=0.41			

* Modified from Azhar * et al.* [23]

The meat chemical parameters of *Aceh* cattle with different *MSTN genotypes* are presented in Table-5. The data showed that meat chemical parameters analyzed were not markedly different among the cattle carry certain *MSTN genotypes*, showing that the chemical parameters of *Aceh* cattle meat were not influenced by *MSTN* gene.

**Table-5:** Average meat chemical parameters of *Aceh* cattle with different *MSTN* genotypes.

Meat chemical parameters	Commercial cut	*MSTN* genotype		

		AA	AB	BB
Water (%)	Sirloin	70.57±4.05^ns^	68.57±5.34^ns^	71.17±3.31^ns^
	Silverside	70.93±5.97^ns^	70.32±4.14^ns^	73.93±4.80^ns^
Ash (%)	Sirloin	0.94±0.09^ns^	0.98±0.09^ns^	0.95±0.12^ns^
	Silverside	0.95±0.12^ns^	0.97±0.09^ns^	0.98±0.11^ns^
Protein (n×6.25%)	Sirloin	18.13±0.31^ns^	17.10±2.56^ns^	16.02±3.12^ns^
	Silverside	18.13±1.72^ns^	17.51±2.72^ns^	17.07±3.41^ns^
Fat (%)	Sirloin	5.83±4.72^ns^	3.74±2.82^ns^	2.28±1.18^ns^
	Silverside	2.26±2.25^ns^	3.09±1.86^ns^	1.59±0.65^ns^
Cholesterol (%)	Sirloin	63.60±22.39^ns^	75.81±16.54^ns^	67.82±16.13^ns^
	Silverside	61.27±23.18^ns^	70.72±15.51^ns^	63.63±16.66^ns^

ns=differences presented in the same row were not significant

## Discussion

Meat is a complex biological system made of water up to 75% that is in balance circumstance with 20% protein, 2% fat, and 3% other small components such as minerals, phosphoric containing molecules, and vitamins [25]. Water, ash, protein, and fat contents of sirloin (GM muscle) and silverside (BF muscle) meats of female *Aceh* cull cattle obtained in this study (Table-2) were different from those reported in other cattle, either *B. indicus* or *B. taurus*. The moisture composition of sirloin and silverside meats of *Aceh* cattle, 69.12±4.50% and 69.95±3.76%, respectively, is lower than that reported in *Bali* cattle meats (72.84±0.79%) [26], GM and *Longissimus dorsi* (LD) muscles of adult *Limousin* x *Luxi* crossbreed steers, 74.4% [27].

Comparing the meats from *Bali* (GM 1.13%-2.15%) [24] and *Limousin* x *Luxi* crossbreed cattle (GM 1.77% and LD 1.66%) [27], the Aceh’s meat contains, in the same muscles, less ash 1.00±0.06% and 1.00±0.06% (GM and LD, respectively). While the protein content of the *Aceh* cattle meats (GM muscle 17.76±1.73% and BF muscle 18.28±1.92%) is in agreement with those reported by Buckle *et al*. [28], 16-22%; they are lower than those found in *Bali* cattle meats, protein 21.64±1.08% [26] or GM and Ld muscles of adult male *Limousin* x *Luxi* crossbreed (22.9% and 22.8%, respectively) [27].

In addition to water, ash, and protein, the fat content of the sirloin and silverside meats of *Aceh* cattle found in this study, 3.53±2.64% and 2.77±1.81%, respectively, were in agreement with the range reported in *Aceh* cattle, 3-6% [29] and male *Limousin x Luxi* crossbreed (1.7%) [27], but markedly lower than that found in *Bali* cattle, 13.82-19.05% [26]. The cholesterol content of *Aceh* cattle meats, GM 76.59±14.78 mg/100 g and BF 72.69±13.23 mg/100 g, was comparable to those reported in the *semi membranosus* and BF muscles of indigenous cattle in southern Brazil measured by enzymatic (60.63±2.33 mg/100 g and 63.02±3.62 mg/100 g, respectively) and HPLC (51.97±1.40 mg/100 g and 63.44±3.75 mg/100 g, respectively) methods [30]. No information is available about the cholesterol content of both *Bali* and *Limousin* x *Luxi* crossbreed cattle to make a comparison.

Khasrad *et al*. [31] reported that cattle breed significantly affected water, protein, and fat contents of the LD muscle of *Bali*, *Pesisir*, *Simmental* cross, and *Brahman* cross cattle. The average water content of meat in *B. indicus* 77.50±0.40% [32] is higher than that in *B. taurus*, 72.40-74.80% [31]. Evidence for the influence of breed on chemical content of meat is also shown by a higher percentage of meat ash of *B. taurus* compared to that of *B. indicus* [32].

The effect of cattle age on meat composition is not only shown by a decreased meat fat content according to age of the *Bali* cattle but also from the fact that the meat of young (2.0-2.5 years old) and old (7-10 years old) *Bali* cattle has significantly lower ash content than that of adult (3.5-6 years old) *Bali* cattle [26]. In addition, the effect of age on the profile of meat chemical parameters was also seen in 1- and 2-year-old *Limousin* x *Luxi* crossbreed cattle [27].

Data in Table-2 also show that the differences in protein, fat, and cholesterol contents between sirloin and silverside meats were not significant. This indicated that different commercial cuts did not have an obvious effect on the chemical composition of the meat of the *Aceh* cattle evaluated. This finding is different from that reported by Rhee *et al*. [33], who found that muscle types have a great effect on the chemical traits of beef meats when analyzed palatability and biochemical traits variation in 11 beef meats. Wang *et al*. [27] also found an effect of muscle types on the nutrient profile of *Limousin* x *Luxi* crossbreeds.

The occurrence of naturally different hair colors might have some benefits in *Aceh* cattle. The results of this study did not show the effect of hair color variations on chemical parameters of *Aceh* cattle (Table-3). Although chemical parameter differences were observed among *Aceh* cattle with different hair colors, they were not significant. Some factors that might contribute to these facts are that relatively high individual variations existed in some chemical parameters among the cattle and the small sample size. Further study must be done to confirm this prediction.

As one of the potential genes that influence the muscle growth and the depth of intramuscular fat in some cattle [13], it is possible that *MSTN* polymorphism affects the chemical composition of meat, a hypothesis that we wanted to analyze in this study. We found the polymorphic condition and balance distribution of *MST*N in *Ace*h cattle, situations that are in agreement with the findings reported in *Bali* cattle by Nugroho *et al*. [18]. Dunner *et al*. [34] also reported a breed-specific haplotype in the *MSTN* of many European cattle breeds. The monomorphic condition of the MSTN genotype, on the other hand, was observed in several European [35,36], Indian [7], and native Turkish breed cattle [37]. The presence of three (AA, AB, and BB) genotypes in *Aceh* cattle (this study) was different from the finding of Nugroho *et al*. [18] showing only AB and BB genotypes identified in *Bali* cattle.

The RFLP digestion using the HaeIII restriction enzyme on *Aceh* cattle *MSTN* performed in this study resulted in flank cut 1346-bp B fragment into 700, 646, 618, and 28 bp fragments. Nugroho *et al*. [18] interestingly found five fragments 90, 100, 250, 450, and 546 bp fragments from the digestion of the 1346-bp *MSTN* of *Bali* cattle amplified using the same primer set. These facts show that different numbers of restriction sites of HaeIII enzyme are available in *MSTN* between *Bali* and *Aceh* cattle.

**Figure-3:**
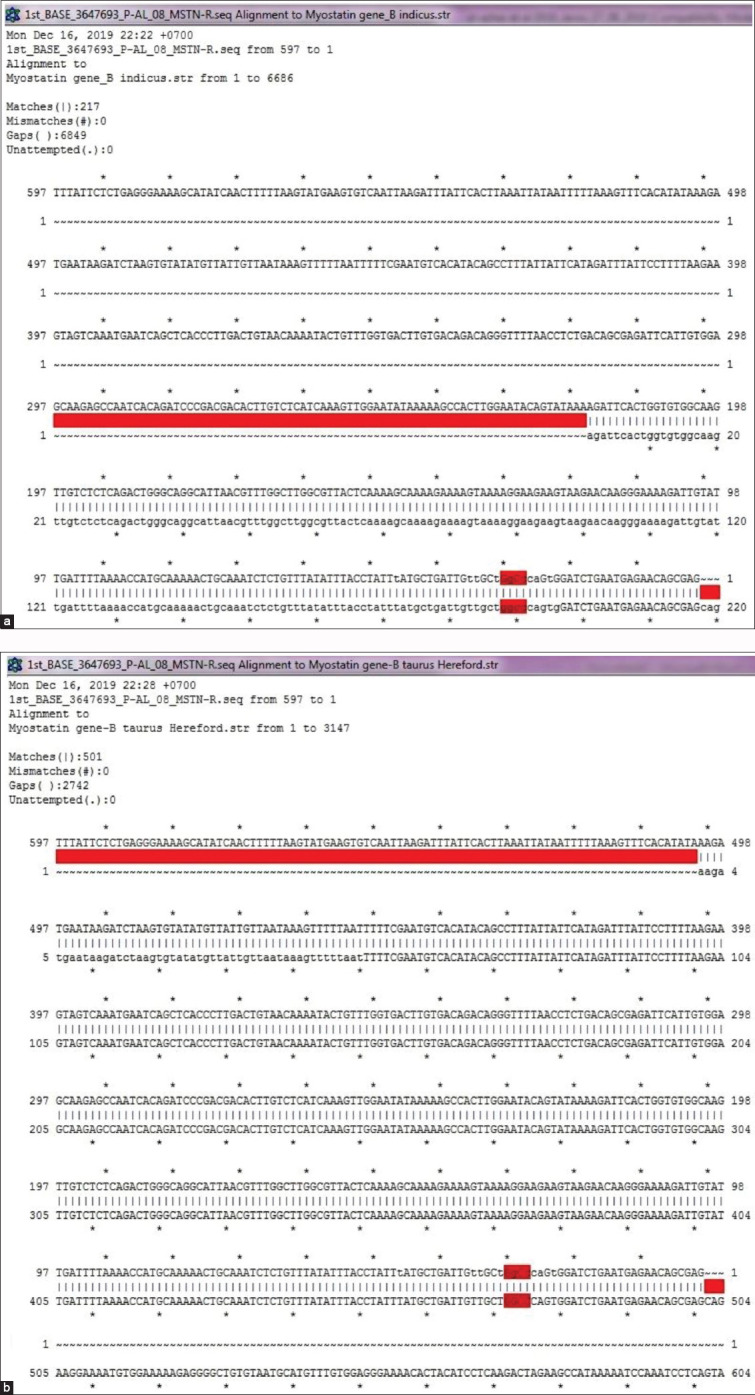
Sequence alignment of *Aceh* cattle *myostatin* (*MSTN*) gene fragment amplified using forward primer and *Bos indicus* *MSTN* gene (a) and *Aceh* cattle *MSTN* gene fragment amplified using reverse primer and *B. indicus MSTN* gene (b) (a) sequence alignment of *MSTN* gene *B. indicus* and *Aceh* cattle (b) Sequence alignment of *MSTN* gene *Bos taurus* and *Aceh* cattle.

To confirm the difference, *MSTN* amplified from one genomic DNA sample with AB genotype (its *HaeIII* digestion resulting in 700, 646, 618, and 28 bp fragments) was sent to PT Genetika Science Indonesia for a commercial sequencing service. Nucleotide sequences of *Aceh* cattle *MSTN* obtained from sequencing using forward and reverse primers were then individually analyzed using APE Software (University of Utah, Salt Lake City, UT). The results showed the absence of a HaeIII restriction site along the 556 nucleotide sequence product of *Aceh* cattle *MSTN* sequenced using forward primer. The 597 nucleotide sequence resulting from *MSTN* sequencing using a reverse primer, on the other hand, contained one HaeIII restriction site at the nucleotide position of 53 (Figure-3). The occurrence of another HaeIII restriction between nucleotide 556 and 749 is assumed, thus confirming the possible occurrence of 1-2 HaeIII restriction sites along the 1346 bp of the *MSTN* fragment of *Aceh* cattle shown by the results of PCR-RFLP analysis. This is a novel finding which shows the specific number and locations of HaeIII restriction sites in the *MSTN* gene *Aceh* cattle.

Deeper analysis on the HaeIII restriction sites along bovine *MSTN* was performed by comparing *MSTN* sequences of *Bos Taurus* Hereford (3147 bp, gene ID: 281187) and *B. indicus* (6686 bp, accession number AY794986), downloaded from GenBank database. The results showed the occurrence of 6 HaeIII restriction sites along the *MSTN* of *B. taurus*, which resulted in seven gene fragments after digestion, namely, 1622, 421, 404, 218, 215, 94, and 23 bp [23]. The *MSTN* of *B. indicus*, interestingly, has nine HaeIII restriction sites, three of them in intron 1 (2 sites) and two that resulted in ten gene fragments after digestion. The result of sequence alignment analysis showed that *Aceh* cattle *MSTN* was aligned with the nucleotide (nt) 1 – 217 of *B. indicus MSTN* and nt1 – nt501 of *B. taurus MSTN* sequences deposited in the GenBank (Figure-3a). The rest of the 1129 and 845 nucleotide sequences of *B. indicus* and *B. taurus MSTN*, respectively, are located on the upper part of the sequences. There is no sequence available to make a comparison between the *MSTN* sequences amplified by forward primer. Altogether, the data presented in this study show that *MSTN* is highly variable in Indonesian beef cattle. This finding is in agreement with the condition found in several cattle, where five out of nine mutations identified in the *MSTN* are located in coding sequences [38]. The different size of DNA fragments, however, indicates possible species specificity of HaeIII restriction sites in cattle. Further PCR experiments to get enough DNA products for sequencing are now performed in our laboratory to provide better results and analysis.

The effect of the *MSTN genotype* on meat chemical parameters of *Aceh* cattle is presented in Table-5. The invariant genotype of *FABP4* found in *Aceh* cattle showed no possible effect of the gene in the chemical composition of the cattle meat, meaning it is not a potential marker for beef production in *Aceh* cattle. The data show meat moisture, ash, protein, fat, and cholesterol contents were not influenced by genotype during the current research trial. Although slight variation existed in meat chemical levels between cattle with the AA, AB, and BB *MSTN genotypes*, the differences were not significant (p>0.05). The potential effect of certain *MSTN* mutation on meat chemical content is shown by reduced external and internal intramuscular fat deposition in cattle carrying a single mutant allele from *Belgian Blue* or *Piedmontese sire* crossbreed compared to the pure breed [39]. These results suggest that selection for meat chemical parameters profile in *Aceh* cattle should be done not only based on *MSTN/HaeIII* variation but also other potential genes influencing meat composition such as calpain and calpastatin, the genes have been known and extensively studied their polymorphisms relationship with meat quality in cattle [40,41].

## Conclusion

The presence of different HaeIII restriction sites of *MSTN* gene, but not *FABP4* gene, is responsible for the polymorphism of genes in *Aceh* cattle. The frequency of *MSTN* *genotype* agreed well with Hardy-Weinberg equilibrium, showing a balanced inheritance of the gene in the *Aceh* cattle population. The study showed that meat chemical parameters are influenced by breed but not by different commercial cuts and *MSTN* or *FABP4* *genotypes*. This suggests that either *MSTN* or *FABP4* is not a potential gene to be used for meat quality related-molecular assisted selective breeding in *Aceh* cattle.

## Authors’ Contributions

AA, MA, and MS designed and performed the experiments. MH and TSR extracted DNA from the samples and were responsible for PCR-RFLP analysis. MS and MH analyzed the data. AA, MA, and TSR wrote the manuscript. All authors read and approved the final manuscript.

## Data Availability

Supplementary data can be available from the corresponding author.
